# EDA, PPG and Skin Temperature as Predictive Signals for Mental Failure by a Statistical Analysis on Stress and Mental Workload

**DOI:** 10.1109/OJEMB.2024.3515473

**Published:** 2024-12-11

**Authors:** G. Luzzani, I. Buraioli, G. Guglieri, D. Demarchi

**Affiliations:** ^1^ Department of Mechanical, Aerospace EngineerPolitecnico di Torino 19032 10129 Turin Italy; ^2^ Department of Electronics, TelecommunicationPolitecnico di Torino 19032 10129TurinItaly

**Keywords:** Biosignals, human factors, mental workload, statistical analysis, stress

## Abstract

*Objective:* The growth of autonomous systems interacting with humans leads to assessing operators' stress and mental workload (MWL), especially in safety-critical situations. Therefore, a system providing information about the psychophysiological workers' condition is fundamental and still missing. This paper aims to study the statistical relationship between the variation of Photoplethysmogram signal (PPG), Electrodermal Activity (EDA), and skin temperature with respect to stress and MWL levels, assessed through an ad-hoc developed subjective questionnaire. *Results:* 43 features were calculated from these signals during the execution of two cognitive tests and processed through a statistical analysis based on Kruskal-Wallis and Mann-Whitney U tests. This analysis proved that about 50% of them offered statistical evidence in differentiating relaxed and altered emotional conditions. Moreover, fifteen features were found to provide sufficient information to detect at the same time stress and MWL. *Conclusions:* These results demonstrate the feasibility of this approach and push to continue this research about the relationship between physiological signals and the variation of stress and MWL by enhancing the population and considering more biosignals.

## I. Introduction

Human Factors and Ergonomics (HFE) is a research area gaining increasing importance in our everyday lives [1], [2]. This discipline applies psychological and physiological principles to designing and engineering products, services, or systems [3]. Since the development of recent technologies involving a more consistent interaction between humans and autonomous machines, also in safety-critical environments, evaluating Mental WorkLoad (MWL) and stress of operators during their jobs is becoming an essential element [4], [5]. However, these terms still need a well-established definition due to their complex and multifaceted nature. In fact, over the years, several researchers have described the concept of MWL by providing different explanations related to their specific application fields [6], [7]. Nevertheless, a recent study by Van Acker et al. [8] tried to find common ground by defining this term as *a subjectively experienced physiological processing state, revealing the interplay between one's limited and multidimensional cognitive resources and the cognitive work demands being exposed to.* Whereas, with respect to the concept of stress, the World Health Organization defined this condition as a state of worry or mental tension caused by a difficult situation, and it is a natural human response that prompts us to address challenges and threats in our lives [9]. These two states could not be considered as two separate entities. In fact, according to the Debie et al. model [6], a direct cause-effect link exists between stress and MWL by seeing the former as a depletion factor that affects the MWL of an operator. The literature shows mainly three ways to assess operators' stress and MWL: subjective evaluations, behavioral analysis, and physiological measures [10], [11], [12]. In particular:

*a) Subjective evaluations:* involve post-performance questionnaires asking operators about their perception of MWL and stress, commonly using tools like NASA TLX, Cooper-Harper, or Bedford scales [13], [14], [15], [16].

*b) Behavioral measures:* involve observing workers' actions and comparing them with a predefined plan, inferring cognitive workload based on errors or missed activities [17], [18].

*c) Physiological measures:* analyze physiological signals (e.g., heart, skin, eye, brain activity, respiration, skin temperature, muscle activation, voice patterns) to assess cognitive states. The growing biomedical sensor market enables new technologies, although a comprehensive, reliable solution is still lacking [4], [6], [19], [20], [21], [22]. In fact, the literature does not agree on the most significant signals for assessing these conditions due to their context-dependent factors, varying definitions, and diverse measurement tools (e.g., NASA-TLX, Bedford Scale). This inconsistency highlights the complexity of measuring MWL and stress, presenting opportunities for further research and methodological standardization [12].

Therefore, our work investigates the correlation between an altered cognitive state (linked to an inducted rise of both MWL and stress) and three of the most relevant physiological signals (already known in the literature). In particular, the chosen were heart activity through the Photoplethysmogram (PPG) signal, skin activity with the Electrodermal Activity (EDA) analysis, and skin temperature [23], [24], [25], [26]. The selection prioritizes easily accessible and unobtrusive physiological signals from localized areas like the hand, enabling applicability in future scenarios. Current wearable devices (e.g., bracelets, rings) demonstrate feasibility by integrating multiple physiological signals, supporting simultaneous data acquisition [27], [28]. However, a robust framework for analyzing stress, MWL, and associated signals is still absent.

**Figure 1.**
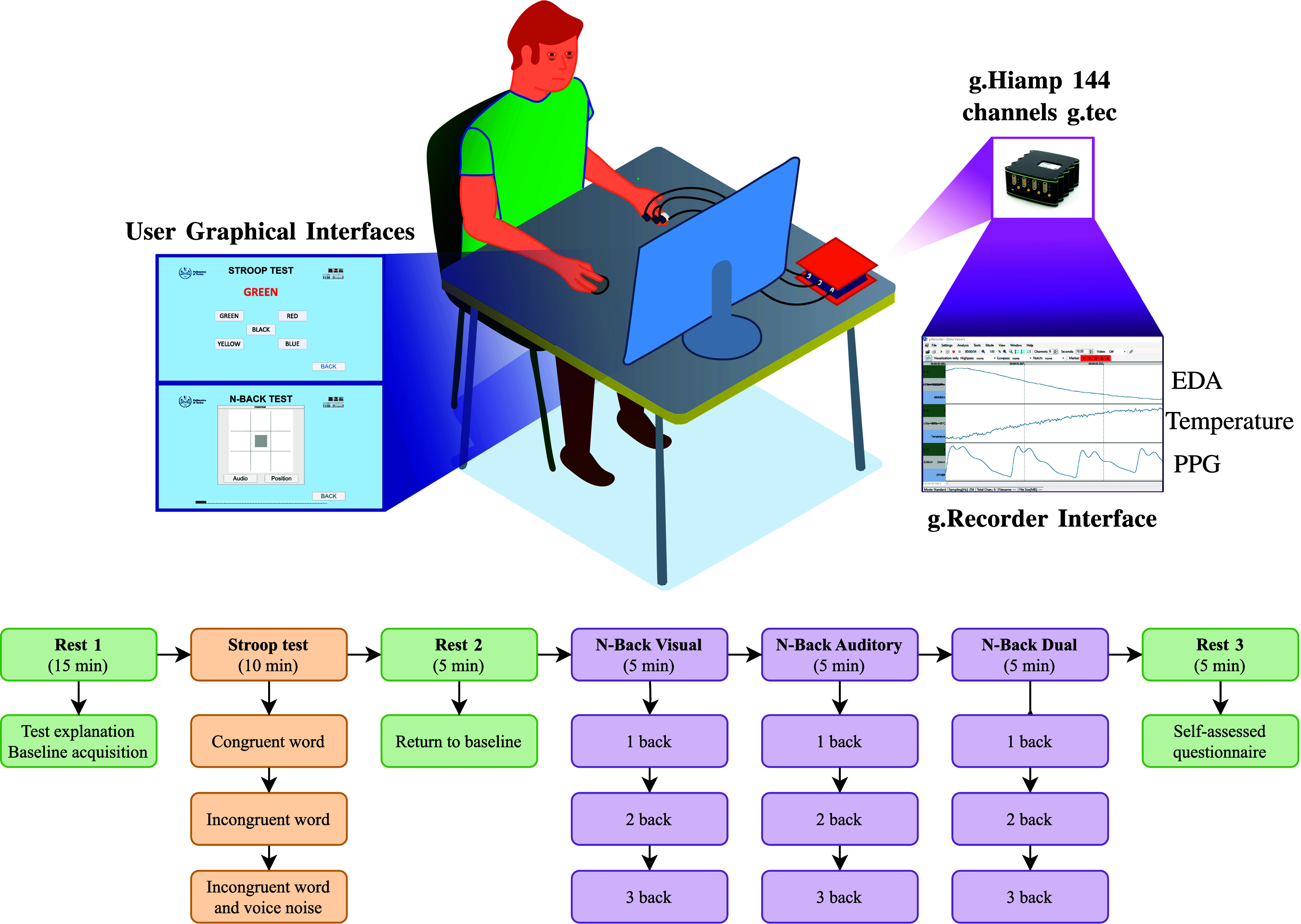
Test procedure adopted for each participant. The different colours highlight the Rest, Stroop, and N-Back phases. The duration of each subphase is also reported. A graphical representation of the test setting with the equipment and the user graphical interfaces is also reported.

A study was conducted with 28 volunteers, approved by the Politecnico di Torino - Ethics Committee (Protocol Number 1606). Participants underwent two cognitive tests: Stroop [29] and N-Back [30], shown in Fig. 1. The Stroop test simulated increasing external stress conditions in three sub-phases, while the N-Back test induced visual, auditory, and dual MWL, each with three increasing difficulty levels (1/2/3-Back). Afterward, participants completed the *Self-Assessment Questionnaire (SAQ)*, rating each phase on a four-level scale to quantify cognitive alteration. PPG, EDA, and skin temperature signals were analyzed, resulting in 43 normalized features per test sub-phase, linked to subjective feedback (Table 2) [31].

**TABLE 1:** Results Obtained Through the Statistical Procedure Shown in Figure 3

		Statistical results			
		**Stroop**	**Visual N-Back**	**Auditory N-Back**	**Dual N-Back**
Overall Analysis OA	**Test Significance**	31	33	26	24
	**Feature Significance ($>1$)**	29	32	24	21
	**Feature Significance ($>2$)**	28	27	21	19
	**Feature Significance ($>3$)**	25	23	20	18
	**Feature Significance ($>4$)**	11	8	4	4
	**Feature Significance ($>5$)**	6	5	0	2
	**Feature Significance ($>6$)**	2	0	0	2
	**Feature Significance ($>7$)**	2	0	0	0
	**Feature Significance ($>8$)**	0	0	0	0
	**Feature Significance ($>9$)**	0	0	0	0
Binary Analysis (BA)	**Test Significance**	31	33	26	24
	**Feature Significance ($>1$)**	29	29	23	18
	**Feature Significance ($>2$)**	27	24	19	18
	**Feature Significance ($>3$)**	24	20	17	18

For Each Dataset (Represented in the Columns), the Number of Resulting Features Was Significant Through Our Test are Reported for Both Overall Analysis (OA) and Binary Analysis (BA).

**TABLE 2:** PPG, EDA, and skin Temperature Features Adopted in Our Analysis

Features Comparison					
**Signal**	**Feature**	**Stroop**	**Visual N-Back**	**Auditory N-Back**	**Dual N-Back**
PPG	Mean amplitude				
	St. Dev. amplitude	•	•		•
	Median amplitude				
	Mean duration	•			
	St. Dev. duration	•	•	•	•
	Median duration	•			
	Mean rise time				
	St. dev. rise time	•	•	•	•
	Median rise time				
HR	Mean BPM	•			
	St. Dev. BPM		•	•	•
	Median BPM	•			
HRV	pNN50	•	•	•	•
	Mean PLF IBI				
	St. Dev. PLF IBI	•	•	•	•
	Mean PHF IBI			•	
	St. Dev. PHF IBI	•	•	•	•
	Mean PLF/PHF IBI			•	•
	St. Dev. PLF/PHF IBI	•	•	•	•
SCL	Mean	•	•	•	•
	St. Dev.	•	•	•	•
	Slope				
SCR	Mean amplitude				
	St. Dev. amplitude				
	Mean rise time				
	St. Dev. rise time	•			
	Average peaks number				•
Temperature	Initial value				
	Final value	•	•		
	Delta value	•	•	•	•
	Mean	•			
	St. Dev.	•	•	•	•
	Variation over time	•	•		
	Variation over time slope		•		
Temperature First Derivative	Initial value	•	•	•	•
	Final value				
	Delta	•	•	•	•
	Mean	•	•		
	St. dev.	•	•	•	•
	Variation over time				
	Variation over time slope	•	•	•	•

For Each Characteristic Marked With a •, the Features That Demonstrated Significant Differences in the Mann-Whitney Paired Comparisons Between Class 0 and the Other Classes are Highlighted. The Features Described in the PPG Section Refer to the Shape Characteristics of the PPG Waves. Heart Rate (HR) and Heart Rate Variability (HRV) are Associated With the Temporal and Frequency Aspects of Beats Per Minute (BPM) Dynamics. Skin Conductance Level (SCL) and Skin Conductance Response (SCR) Relate to the Slow and Fast Components of the EDA, Respectively, With SCR Features Specifically Reflecting the Waveform Characteristics of This Signal. Additionally, the Characteristics of Temperature and Its First Derivative are Detailed. Further Information Can Be Found in Section V and in the Supplementary Materials.

The primary goal of this research is to assess the feasibility of investigating the relationship between PPG, EDA, and skin temperature, and perceived stress or MWL, using the simplified *SAQ*. This tool, designed for ease of use in computerized tests and industrial scenarios, offers reduced complexity compared to NASA-TLX and Bedford scales.

Our research introduces novelty in the following ways:

1) We analyzed physiological responses to externally induced stress and cognitive workload using tasks like Stroop and N-Back tests, assessed with the *SAQ* (detailed in the *Supplementary Materials*).
2) The *SAQ* independently evaluates stress and MWL, making it more practical for computerized and industrial applications, and facilitates feature clustering for machine-learning-based algorithms.
3) We focused on wearable-friendly, non-invasive signals that can be easily collected from a small body surface area, minimizing intrusion for real-world applications.

Statistical analyses were conducted to identify which features of these physiological signals are significant in discriminating subjective perceptions of stress and MWL.

## II. Results

Our data analysis considered the tests separately, leading to four different datasets: Stroop, N-Back Visual, N-Back Auditory, and N-Back Dual. As shown in Fig. 1, the four tests were divided into three phases. Thus, considering an additional *Rest phase* calculated on the physiological data acquired during the initial relax part, each dataset comprised the same amount of data:

\begin{equation*}
n_{data} = n_{participants} \times n_{phases} \times n_{features} = 4816 \tag{1}
\end{equation*}

where $n_{data}$ was the overall number of data of each test dataset, $n_{participants}=28$ was the number of participants, $n_{phases}=4$ was the number of phases for each test, and $n_{features}=43$ was the number of features. These data distributions were standardized through a maximum-minimum normalization process applied to each subject's physiological characteristic.

**Figure 2.**
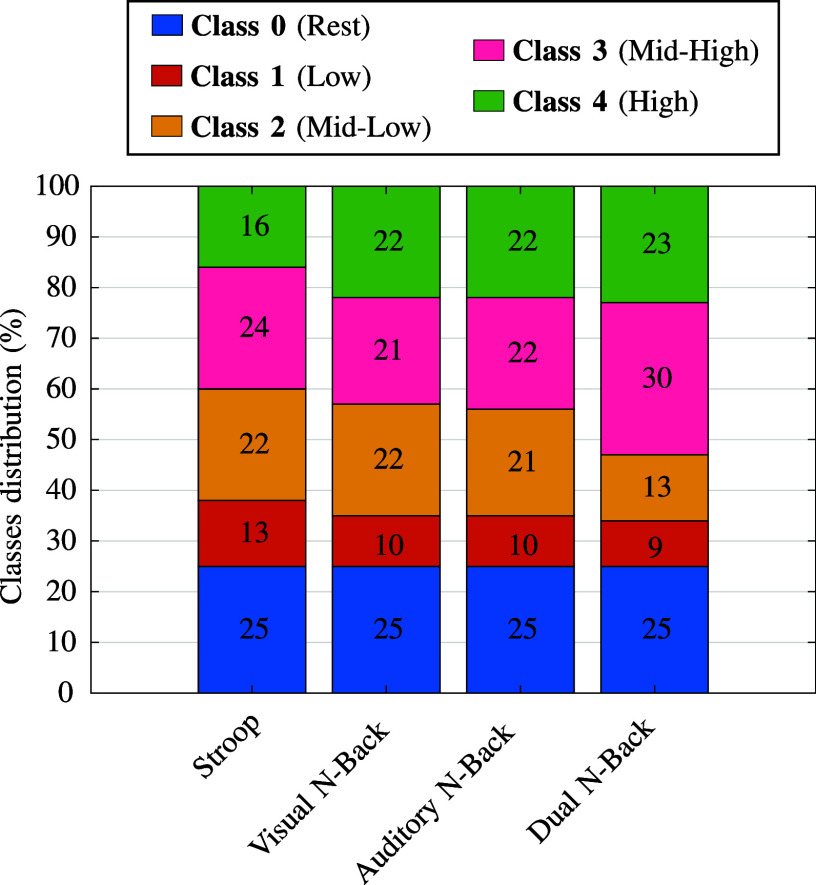
Percentage population distribution for each test modality (Stroop, Visual N-Back, Auditory N-Back, and Dual N-Back) across the four levels of perceived stress (associated with the Stroop) or MWL (linked to the N-Back), namely Class 1 (Low) to Class 4 (High). Additionally, Class 0 represents the baseline values acquired during the initial Rest phase. This distribution is based on the outcomes of the Self-Assessment Questionnaire administered to participants at the end of the procedure.

**Figure 3.**
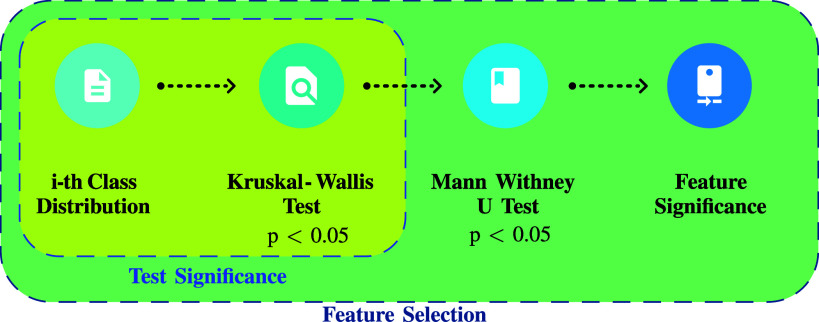
Data analysis statistical procedure adopted to evaluate the significance of the features concerning the variation of MWL and stress.

**Figure 4.**
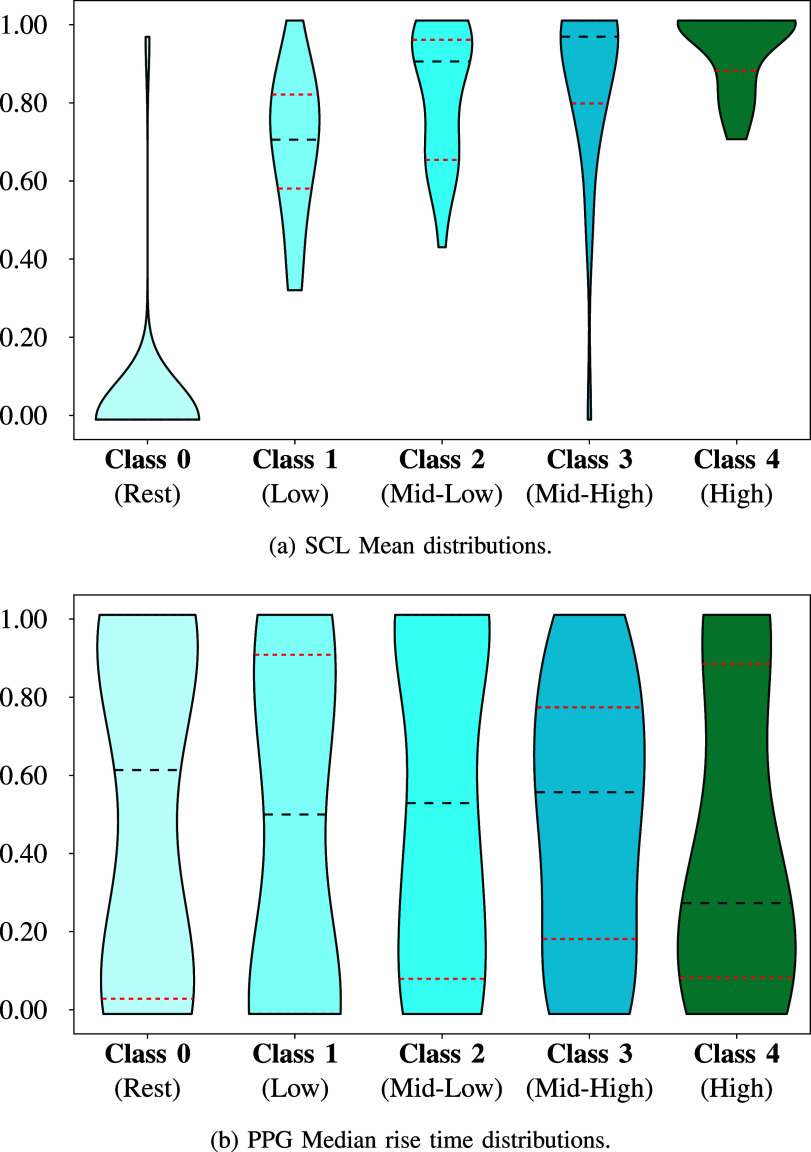
This Figure shows two different conditions that we want to differentiate in our analysis. In Fig. 4(a), the difference between the relaxed condition (Class 0) and the cognitively altered ones is evident, while in Fig. 4(b), it is evident how it is impossible to observe changes between the five classes. We want to assess these aspects through our statistical analysis process, which results are reported in Table 2.

To evaluate if *each feature* underwent variation according to the different cognitive states, the statistical analysis described in Fig. 3 was implemented. It was structured in three steps:

1) For each test phase, we matched the 43 physiological characteristics of the subjects with their corresponding answers on the four-level scale of the *SAQ*, which was administered at the end of the test. Additionally, we introduced a class named *Class 0* to each dataset, representing the features measured during the initial relaxed condition (**Rest 1** in Fig 1). This class reflected the baseline values, as these data were only recorded during this phase. The **Rest 2** and **Rest 3** phases were used to restore signals to baseline levels after the testing efforts. For this reason, from now on in the paper, the terms “relaxation,” “baseline,” and “relaxed” will be used interchangeably to refer to the values associated with *Class 0*.
  The final percentage allocation in the resulting five labels (from 0 to 4) is reported in Fig. 2. It is evident how this is a non-homogeneous distribution because we want to investigate the variation of physiological data from a personal perspective; that is the one we retain as being the most effective for studying subjective conditions like MWL or stress.
2) Since it was impossible to assume a normal condition for each dataset class, it was decided to implement the non-parametric Kruskal-Wallis (K-W) test [32]. As shown in Fig. 4, it inputed the distributions associated with the labels, providing information about their differences. In particular, if the *p-value* resulted in $p< 0.05$, it followed that at least one distribution was statistically different. Fig. 4(a) reports the SCL Mean example where it is clear how the Class 0 (relax condition) is extremely different from the other classes representing the altered cognitive states. Instead, Fig. 4(b) depicts the condition where it is impossible to find statistical differences among the classes. This strategy allowed us to demonstrate the dependency of the physiological characteristics involved in the variation of cognitive states.
3) To conduct a more thorough analysis of the significance of each feature, we used the Mann-Whitney U (M-W) test [33]. To reduce false positives from this multiple comparison analysis, we used the Benjamini-Hochberg correction with a false discovery rate of 0.05 [34]. The idea was to adopt a correction algorithm without being too restrictive due to the explanatory and preliminary nature of this study.

The last step of this procedure was applied in two different modalities: *Binary Analysis (BA)* and *Overall Analysis (OA)*. The former aimed to understand which feature was sensitive to the variation of external cognitive workload or stress with respect to a rest condition, therefore performing the M-W between the population of Class 0 and each of the other four classes (in particular, Class 0vs Class 1, Class 0vs Class 2, etc.). The latter represented a deeper analysis, including pairs related to the cognitively altered conditions (Class 1vs Class 2, etc.).

The results of both BA and OA analysis are reported in Table 1. The first row (**Test Significance**) indicates the number of characteristics with a $p< 0.05$ in the K-W test. The other rows (**Feature Significance ($>$n)**) show the features that obtained a statistical significance in at least $n+1$ M-W paired comparisons. To demonstrate the sensitivity of PPG, EDA, and skin temperature in detecting different levels of perceived stress and MWL as assessed by the *SAQ*, we present the overall results of this analysis in Table 1. Detailed comparisons, which are beyond the scope of this study, are not included. In fact, it is important to highlight that Table 1 highlights the analysis of statistically significant features for distinguishing cognitive states. Unlike traditional methods focused on general trends, this study assesses each feature's ability to differentiate five MWL and stress classes, providing a targeted evaluation of sensitivity, reliability, and practical applicability.

Moreover, the full list of the selected features that resulted significantly in all four M-W paired comparisons in the BA analysis (the fourth row of BA in Table 1) for each dataset is indicated through a bullet (•) sign in Table 2, showing how each of the considered signals provided useful features to discriminate stress and MWL from relax.

## III. Discussion

The discussion of the results in Section II is structured into three distinct parts. First, the results presented in Table 1 are analyzed. Second, a comparative analysis of the features detailed in Table 2 is conducted. Finally, this section concludes with a comprehensive assessment of the research findings.

### A. Statistical Analysis Discussion

Observing the OA of Table 1, datasets show a sharp drop in significant features after the fourth row, reflecting statistical differences across three M-W tests. Stroop and Visual N-Back halve from 25 to 11 and 23 to 8, respectively, while Auditory and Dual reduce further to 4 features. Comparing the first four rows of OA and BA reveals similarities, with a maximum 15% difference in Auditory N-Back (20 to 17 features). This suggests most features differentiate between the baseline (Class 0) and stress/mental workload states (Class 1–4), as shown by BA's last row, where 50% of the initial 43 characteristics demonstrate this sensitivity. Stroop and Visual N-Back retain the highest attributes, with 24 and 20 significant features, but finer distinctions among the four altered states remain challenging. OA's last row confirms no dataset fully differentiates the five states.

Additionally, both OA and BA highlight Stroop and Visual N-Back as most sensitive, suggesting stress and visual MWL impact PPG, EDA, and skin temperature more than auditory and dual MWL. Volunteer feedback indicated Auditory and Dual N-Back were harder to follow, with some abandoning the tasks, potentially stabilizing physiological signals toward baseline and reducing significant features.

### B. Features Comparison Discussion

Considering the features given in the output from our analysis, it is clear how PPG, EDA, and skin temperature provided useful information for each dataset separately and, therefore, for the stress and visual, auditory, and dual MWL evaluation. Nevertheless, an interesting consideration was represented by comparing each subset of features to analyze whether any are in common. As shown in Table 2, fifteen common characteristics define the outcome (the ones with the (•) sign in all four datasets). Concerning heart activity, they are about 30% of the initial set: the standard deviation of the PPG's rise time and duration, the pNN50, the standard deviation of the low and high-frequency content of the beats per minute (BPM), and the standard deviation of the ratio between Power of Low Frequency (PLF) and Power of High Frequency (HLF). The EDA signal provided 2 out of 8 significant common features regarding the SCL mean and the standard deviation of the SCL. Moreover, the temperature resulted consistently in about 40% of the starting characteristics: the delta and standard deviation of the temperature, and its first derivative, relatively the initial value, delta, standard deviation, and slope.

### C. Comprehensive Assessment

These results demonstrate that our signals are sensitive to varying levels of stress as well as to visual, auditory, and dual MWL as assessed through our SAQ. Notably, these data clearly differentiate a baseline condition from an altered state. However, only a few features from the initial set of 43 could partially distinguish among four increasing levels of stress or MWL (Class 1 to Class 4). This reduced sensitivity is likely due to the high number of classes relative to the population size. While these results are promising, they indicate the need for a larger dataset to increase the sample size for each class and to facilitate the application of machine learning or AI-based algorithms.

## IV. Conclusion

This paper explores the relationship between physiological signals (PPG, EDA, skin temperature) and stress, visual, auditory, and dual MWL, using statistical analysis. By applying stress and MWL through Stroop and N-Back tests, significant features were identified, with 50% of initial characteristics differentiating between relaxed and stressed/MWL conditions. Fifteen features were consistently significant across all datasets, demonstrating the potential for simultaneous stress and MWL monitoring. The findings emphasize the feasibility of this approach and call for further research with a larger population and additional biosignals.

In conclusion, this study explored a physiological multimodal approach to advance the development of HMI systems. With the rapid growth of the biomedical sensor market, the availability of smaller, cost-effective, and reliable wearable sensors enables the creation of technologies that were previously unfeasible, enhancing safety and driving the next generation of systems.

## V. Materials and Methods

This section introduces the equipment adopted and the signal processing of PPG, EDA, and skin temperature. We engaged 28 participants on a voluntary base, composed of 64% males and 36% females. The age range is 23 to 41, with a mean of 26 years. The *g.tec* *g.HIAMP 144 Biosignal Amplifier* hardware and the relative *g.Recorder* software were used. The sensors used in this experiment are: *g.GSRsensor2* placed on the second phalanx of fingers 2 and 3 to measure the EDA signal; *g.SENSOR Oxygen Saturation* on the tip of the index finger with the LED above to measure the PPG; *g.SENSOR temperature* to measure the peripheral external body temperature, with the thermo-sensor on the fingertip of the fifth finger. The parameters set during the acquisition of the biosignals were: a sampling frequency of 1200 Hz, a notch filter at 50 Hz, a low-pass filter at 30 Hz to all of them, and a high-pass filter at 0.1 Hz to the PPG.

### A. Signal Processing

Once obtained the PPG, EDA, and skin temperature during the execution of the tests reported in Fig. 1, the signals processing part could start. The objective was to calculate for each participant and each test phase the features reported in Table 2.

#### 1) Heart Activity

**Figure 5.**
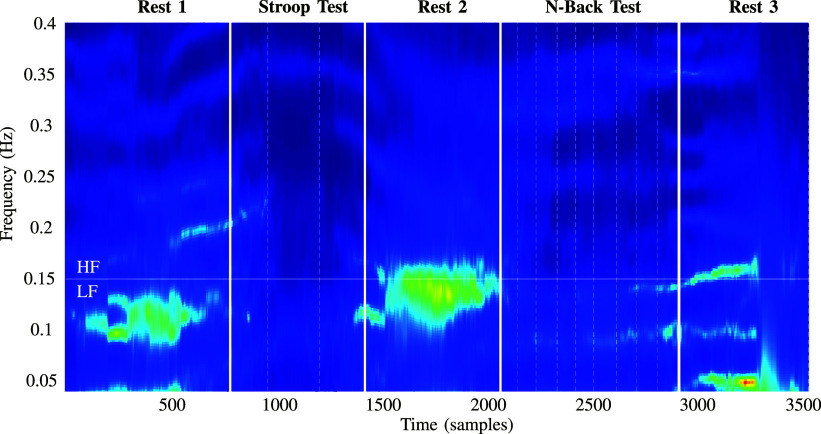
BPM spectrogram of one volunteer during the execution of the tests. The vertical white dashed lines represent the different phases of the procedure, as illustrated in Fig. 1. It is evidence of a change in the power distribution between high and low frequency during the execution of the Stroop and N-Back tests.

It was obtained by analysing the PPG signal. As shown in Table 2, its characteristics refer to the *PPG shape*’s amplitude, duration, and rise time. For each of them the mean, median, and standard deviation in a window of 10 s were evaluated. To assess the heart rate *HR* and heart rate variability *HRV* in the time domain the mean, median, standard deviation, and pNN50 of the beats per minute (BPM) trend were calculated. Moreover, a frequency analysis was also performed by extracting the low (PLF 0.04 Hz to 0.15 Hz) and high-frequency (PHF 0.15 Hz to 0.4 Hz) content from the BPM trend [35]. Finally, the ratio between PLF and HLF was evaluated as a key indicator of stress and mental workload [36], [37]. As reported in the spectrogram in Fig. 5, it is clear how, during the execution of both the Stroop and N-Back test, the frequency content of the BPM trend is changing, providing useful information about the psychophysiological state of a person.

#### 2) Electrodermal Activity

This physiological signal can be divided into slow and fast components [38]. The former, Skin Conductance Level *SCL*, was studied by assessing each test phase's mean, standard deviation, and slope. The latter, specifically Skin Conductance Response *SCR*, was set by evaluating the averaged mean and standard deviation of the amplitude and rise time of the SCR's peaks and their averaged number per phase. Both the noise removal and the separation between SCL and SCR were performed through the cvxEDA method introduced by A. Greco et al. [39].

#### 3) Skin Temperature

Since this physiological signal is not so widely studied in the literature, we decided to assess some features related to its first derivative, never introduced in the analysis of the MWL and stress level [40]. In particular, as presented in Table 2, the values that were evaluated for both the skin temperature raw signal and its first derivative were: initial value, final value, delta (final less initial value), mean, standard deviation, variation over time (delta value over the selected time interval), and variation over time slope (first coefficient of the linear regression of the temperature or its first derivative in the relative phase).

## SUPPLEMENTARY MATERIAL

The Supplementary Material explains the implemented Stroop and N-Back tests and the developed graphical interface. The full procedure of processing PPG, EDA, and skin temperature is also reported. The most significant features from the statistical analysis introduced in Fig. 3 are also reported.

*Ethics Statement:* The Politecnico di Torino Ethics Committee approved our procedure on 13 January 2023 (Protocol Number 1606).

*Conflict of Interest:* The authors declare no conflicts of interest related to this work.

*Author Contributions:* **Gabriele Luzzani:** Conceptualization, Methodology, Data Collection, Software Development, Statistical Analysis, Visualization, Validation, Writing—Original Draft.

**Irene Buraioli:** Conceptualization, Methodology, Visualization, Validation, Supervision, Writing—Review & Editing.

**Giorgio Guglieri:** Resources, Supervision, Writing—Review.

**Danilo Demarchi:** Resources, Supervision, Writing—Review.

Supplementary Materials
