# Supplementary material for: EDA, PPG and Skin Temperature as Predictive Signals for Mental Failure by a Statistical Analysis on Stress and Mental Workload
Source: IEEE Open J Eng Med Biol. 2024 Dec 11;6:248–55. doi: 10.1109/OJEMB.2024.3515473 (PMC11793858; doi:10.1109/OJEMB.2024.3515473)
Supplement: Supplementary Materials [file supp1-3515473.pdf]

Supplementary Materials

EDA, PPG and Skin Temperature as Predictive Signals for Mental Failure by a Statistical Analysis on Stress and Mental Workload

Gabriele Luzzani*, Irene Buraioli, Giorgio Guglieri and Danilo Demarchi

I. Involved physiological signals

THE analysis of subjective perceptions and behavioural observations are two ways to assess MWL and stress that have been studied since the last decades of the twentieth century. Nevertheless, the physiological response to these external stimuli has accelerated recently. The current technological development has yet to define which signals are the most significant, opening up new spaces for research in this area. Therefore, this is the goal of our activity, and this work will focus on the most significant physiological signals according to the literature: the heart and skin activity [1] and the skin temperature. It is important to underline that we aim to consider all those signals that could be implemented in human-machine interface applications. In particular, we decided to monitor all the signals that could be measured on a single hand, considering the unobtrusiveness of the sensors.

A. Skin activity

The monitoring of skin activity is known as electrodermal activity (EDA). This signal is related to the functioning of the sweat glands and the epidermal blood vessels, which are exclusively led by the sympathetic nervous system. Its activation is linked to stress and mental workload reactions [1] by modifying the sweating of the skin and, consequently, the conductance. Thus, it is possible to infer a person's stress or mental workload enhancement by measuring the variations in the electric skin conductance. [2] The EDA signal can be divided into two main phase: a slow tonic component named Skin Conductance Level (SCL) and a fast phasic part defined as Skin Conductance Response (SCR). The former is linked with the slow reaction to external stimuli, while the latter represents the short-time response.

B. Heart activity

Of all the signals that exist to analyse cardiac activity, the most commonly used are the electrocardiogram (ECG) or the photoplethysmogram (PPG) [3]. The ECG represents the projection of the heart's electrical functioning on several derivations, resulting in a signal made by five waves: P, Q, R, S, and T. On the other hand, the PPG signal consists of flooding a vessel underneath with light (typically of infrared wavelength) and measuring changes in the light reflected. The latter is proportional to the amount of oxygen in the vessel, which varies with the pulse and thus allows its progress to be monitored.

Both of these measures allow us to evaluate the peak-to-peak interval of heart activity to assess the heart rate (HR) and the heart rate variability (HRV), which contains significant information about the changes in stress and cognitive workload conditions [4]. It is important to highlight that, during the execution of our tests, we decided to measure HR and HRV through the PPG analysis because it fulfills our requirements (unobtrusiveness and compactness to be placed on a single finger).

C. Skin temperature

The significance of skin temperature in stress and mental workload is linked to the so-called fight-or-flight mechanism. It is a physiological reaction to perceived threats that involves peripheral vasoconstriction, leading to reduced blood flow and temperature in the peripheral limbs, particularly noticeable in the finger [5]–[8]. Therefore, given the hand's susceptibility to temperature changes, we decided that the most optimal placement for the skin temperature sensor would be at the tip of the index finger during our tests.

II. Materials and Methods

This section aims to present all the characteristics of our study, first introducing the involved population, the adopted equipment, and the description of the test. Then, an explanation of the developed procedure and the feature extraction process will be exploited.

A. Participants

We engaged a population selected on a voluntary basis. In particular, we obtained 28 participants, 64% males and 36% of females. The age range is 23 to 41, with a mean of 26 years.

B. Equipment

We decided to employ the hardware and software products provided by the company *g.tec* in collaboration with the *PolitoBIOMed Lab* of the Politecnico di Torino. In particular, we decided to adopt the *g.HIAMP 144 Biosignal Amplifier* as a synchronized multi-channel signal acquisition device. The sensors used in this experiment are:

- *g.GSRsensor2* placed on the second phalanx of fingers 2 and 3 to measure the EDA signal;
- *g.SENSOR Oxygen Saturation* on the tip of the index finger with the LED positioned above to measure the PPG;
- *g.SENSOR temperature* to calculate the peripheral external skin temperature, with the thermo-sensor on the fingertip of the fifth finger (to measure the greater temperature variations than other positions).

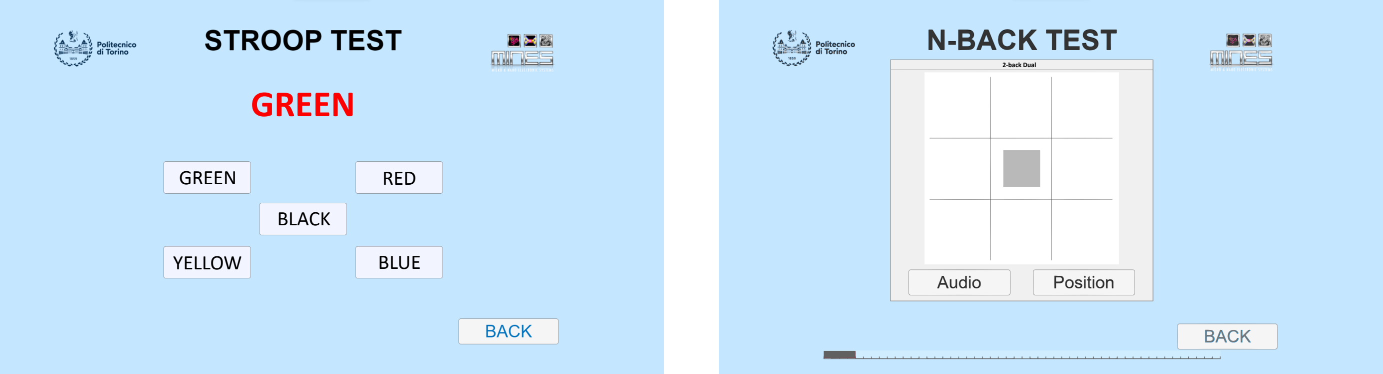

(a) Stroop test graphical interface.

(b) N-Back test graphical interface.

Fig. 1. Computer graphical interfaces adopted during our tests. Figure 1a is related to the Stroop test to stimulate external stress in the participant. Figure 1b shows the N-Back screen implemented to engage different mental workload levels.

The g.HIAMP device interacts directly with the relative *g.Recorder* software, allowing the setting of the sampling frequency and digital filters and real-time monitoring of the biosignals acquired. In particular, we decide to fix a sampling frequency of 1200 Hz, a notch filter at 50 Hz for all of them, and the digital filters reported in Table I.

C. Test

Stress and cognitive load variations were simulated through two specific computerized tests widely studied in the literature: the Stroop [9] and the N-Back tests [10].

a) Stroop Test: This psychological tool is employed to simulate a *stress* condition. It involves displaying a series of words that represent color names in the local language, with the words themselves colored differently. Participants are required to click the button corresponding to the color of the word's ink. The display used during the test is illustrated in Fig. 1a.

b) N-Back Test: This test is designed to induce varying levels of mental workload. Participants are presented with a sequence of stimuli (such as letters, numbers, or locations) and must identify when the current stimulus matches one presented "N" step earlier. The difficulty level is modulated by adjusting the value of N (e.g., 1-back, 2-back, 3-back), with higher values demanding increased working memory capacity and attention to track and recall the sequence. Fig. 1b shows the graphical user interface used in our test.

D. Procedure

This section presents the procedure used in our research to evaluate PPG, EDA, and skin temperature under two specific conditions: stress induced by the Stroop Test and MWL assessed through the N-Back Test. The aim of this approach was to measure stress and workload across visual, auditory, and dual-task scenarios while ensuring that participants returned to a baseline condition before each test.

**Signal**	**Low-pass filter cut-off frequency (Hz)**	**High-pass filter cut-off frequency (Hz)**
EDA	30	-
PPG	30	0.1
Temperature	30	-

TABLE I. Low and high pass filter's cut-off frequencies inserted in the g.Recorder software during the acquisition of the biosignals. The symbol - indicates that the filter was not implemented.

The entire test lasted approximately one hour to complete. The test took place in a room at the Politecnico di Torino with controlled temperature (23 °C) and humidity (50%), without sound disturbance. This was necessary to ensure that the behaviour of the human body was not affected by external stressful agents and that the cognitive phenomena of interest could be specifically studied. Prior to the test, volunteers were provided with comprehensive information about the study procedure and our research objectives. Then, the informed consent and privacy agreements were obtained in accordance with our approved protocol. Participants were instructed to remove watches, rings, and bracelets from their hands and wrists, to turn off their mobile phones, and to refrain from washing their hands immediately before the test to maintain natural skin conditions. This preparatory period, lasting about 10 min, also allowed participants to acclimatize to the room's environment and temperature. Subsequently, each volunteer was seated at a desk in front of a computer, and three sensors were attached to their non-dominant hand (the left hand for all 28 participants). Following these preparations, the procedure detailed in the Scientific Manuscript was initiated.

- **Rest 1**: The initial phase of our test involved the *baseline* acquisition. Participants were instructed to relax completely for 5 min to establish baseline values for their PPG, EDA, and temperature signals. After the baseline acquisition, participants were given an explanation of the Stroop and N-Back Tests, accompanied by a brief demo for practice, lasting approximately 10 min. Following this, an additional 3 min to 4 min was allocated to allow physiological signals to return to baseline levels before initiating the test.
- **Stroop Test:** The Stroop test utilized the incongruence between the color name and the ink color to induce cognitive conflict, resulting in increased response times for correct answers. Due to the brief response time allocated for each

question, this test effectively served as a stress inducer. An additional auditory stressor—a rapidly ticking clock with varying volumes—was introduced to further intensify the stress stimulation. The variable volume prevented the habituation of noise.

The test was divided into three levels of escalating difficulty, every one consisting of 90 questions with a maximum response time of 2.5 s for each. Incorrect answers triggered an acoustic buzz intended to correlate with test performance, thus enhancing stress levels inversely. The experiment was structured into three levels, each separated by a brief 15 s pause to allow participants to prepare for the subsequent phase. These were:

– **Congruent Word:** The meaning of the word displayed was congruent with its color, and the answer buttons were kept in the same position throughout the test.
– **In-congruent Word:** The meaning of the word displayed was in-congruent with its color, and the position of the answer button varied with each question.
– **In-congruent Word + Noise:** In addition to the incongruence between the word and its color and the varying positions of the answer buttons, an audio clip that randomly repeated color names was introduced. This further amplified the cognitive conflict by creating additional discord between visual, reading, and auditory stimuli.

It is important to note that the only instruction provided to participants before the test was to press the button corresponding to the color of the displayed word rather than its meaning. Consequently, they were unaware of the variations between the phases and the presence of the distractors introduced during the test.

- **Rest 2:** A 5 min rest period allowed physiological signals to return to baseline levels. This interval gave volunteers sufficient time to achieve baseline conditions before beginning the N-Back Test and helped to mitigate any potential disturbances in physiological data from the Stroop Test, thus facilitating separate assessments of stress and mental workload. The duration of this rest period was determined by the dynamic response of the physiological signals, with the slower ones, such as EDA and temperature, requiring a few minutes to stabilize. It is important to note that the physiological data collected during this rest phase were not included in the statistical analysis, as this period was solely intended to re-establish baseline conditions between the two tests.
- **N-Back Test:** The N-Back test, detailed in Section II-C, is a cognitive assessment tool designed to measure mental workload. In our procedure, this evaluation consisted of three modalities: auditory, visual, and dual. Every modality was administered across three progressively challenging levels (1-back, 2-back, and 3-back), with 30 questions each. Participants had a maximum of 2.25 s per question [11]. A 5 s relaxation period was provided between each level of complexity, and a 15 s interval was given between different N-Back modalities. These intervals gave participants adequate time to move on to the next phase of the test. The procedure was structured as follows:

  – **Visual N-Back:** As the name says, this modality was designed to induce different levels of *visual mental workload*. Specifically, a square grid consisting of nine boxes was displayed on the screen, as illustrated in Fig. 1b. Within this grid, a grey square moved and changed its position every 2.25 s. Participants were required to press a button with the voice *Position* whenever the grey square returned to the location previously occupied *N* steps earlier (*N* could be 1-back, 2-back, or 3-back).
  – **Auditory N-Back:** This modality aimed to induce distinct levels of *auditory mental workload*. To achieve this, participants were presented with a sequence of randomized English letters. They were required to press the *Audio* button when they heard a letter that matched one presented *N* steps earlier in the sequence, following the same mechanism as the Visual N-Back.
  – **Dual N-Back:** The test concluded with a final series of 1-back, 2-back, and 3-back tasks that integrated both auditory and visual mental workloads, referred to as *dual mental workload*. In this phase, the mechanism of the Visual and the Auditory N-Back tasks were integrated to create a combined cognitive load. Notably, no correlation was established between the two mechanisms, allowing for independent stimulation of the visual and auditory channels.

  Moreover, a red progress bar was added to each modality and positioned at the bottom of the graphical interface. Since this research aimed to assess mental workload, participants were informed about the test procedures in advance, without the inclusion of distractors or stressors, to ensure that the cognitive load could be isolated as accurately as possible.
- **Rest 3:** The experiment concludeded with a final rest phase, during which participants completed a Self-Assessment Questionnaire regarding their subjective perception of mental workload and stress. This tool was deeply described in section II-E.

It is important to highlight that the tasks were not randomized, as our research objective was to examine subjective perceptions of mental workload or stress. For example, if a participant experienced fatigue while performing the N-Back test, this condition did not compromise our analysis since it would be captured through the subjective questionnaire administered at the end of the tasks. The statistical analysis, as detailed in the Science Manuscript, relied on the division of perceived mental workload or stress rather than the order of task presentation.

E. Self-Assessment Questionnaire

A key component of our research was the *Self-Assessment Questionnaire*. The literature has complex subjective workloads

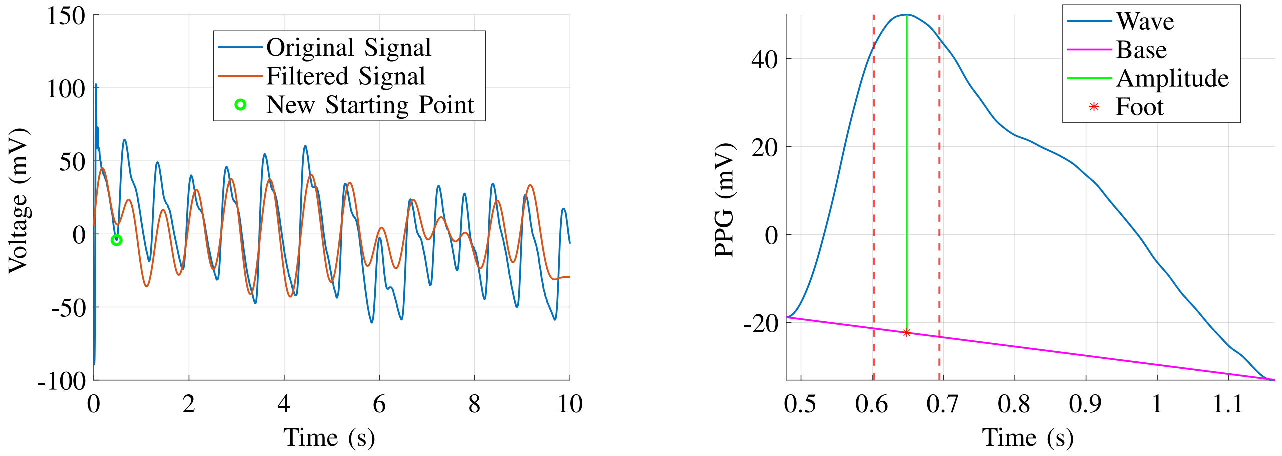

(a) Starting point selection.

(b) PPG's single wave.

Fig. 2. This Figure provides two steps to process the PPG signal. Figure 2a shows the method adopted to obtain an initial starting point. Figure 2b reports the features related to the shape of the PPG signal.

and scales to evaluate stress and mental workload independently, such as the NASA-TLX or Bedford questionnaires. Unfortunately, they were unsuitable for our experiments, forcing us to develop a custom questionnaire tailored to our objectives. Participants were asked to rate each phase of the Stroop and N-Back tests on a four-point scale, with "1" indicating low cognitive alteration and "4" signifying a high level of stress or mental workload, depending on whether the rating pertained to the Stroop or N-Back test, respectively. For the three levels of the Stroop test, the questionnaire focused on assessing *stress*, defined as the perceived discomfort caused by external or internal disturbances, such as background noise or shifting answer button positions. For each phase of the N-Back test (visual, auditory, or dual) the questionnaire assessed the *mental workload* experienced. This cognitive state was characterized by the fatigue associated with managing an excessive amount of information, as represented by the 1-back, 2-back, and 3-back levels, across various channels (e.g., visual, auditory, or both visual and auditory). The four levels of the scale were designed based on the broad categories outlined in the Bedford questionnaire.

F. Feature Extraction

The next step in the pipeline of our work was represented by processing the acquired signal to extract all the features that, from a literature point of view, showed a relationship between the variation of stress and cognitive workload. The code was developed in a *Matlab* environment.

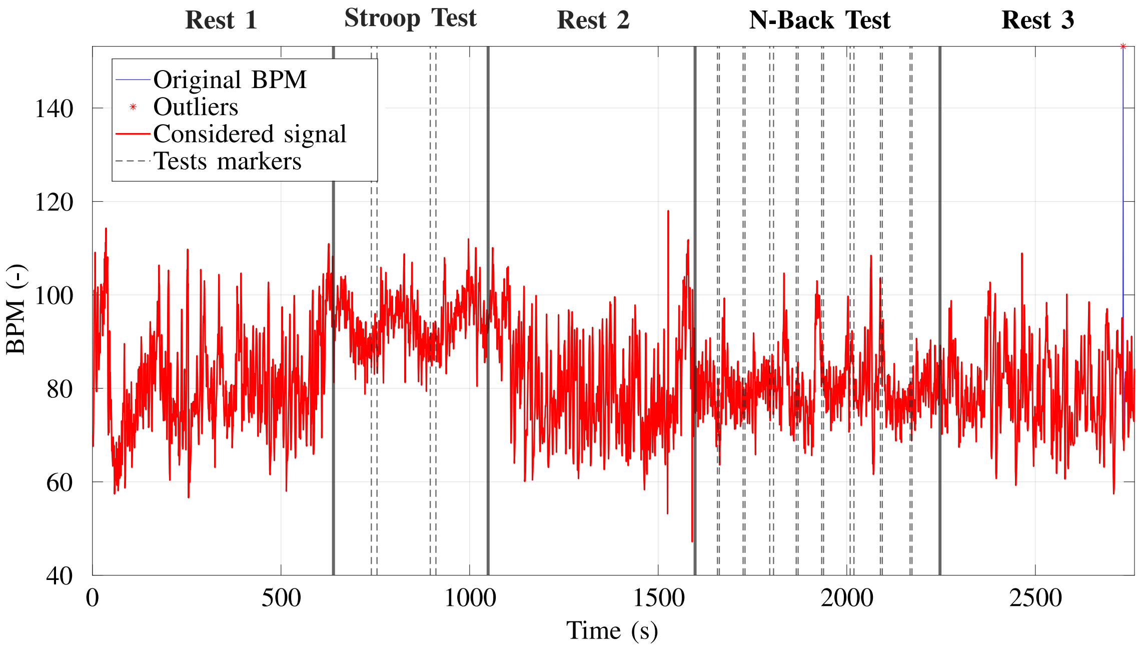

Fig. 3. BPM output obtained from the PPG process.

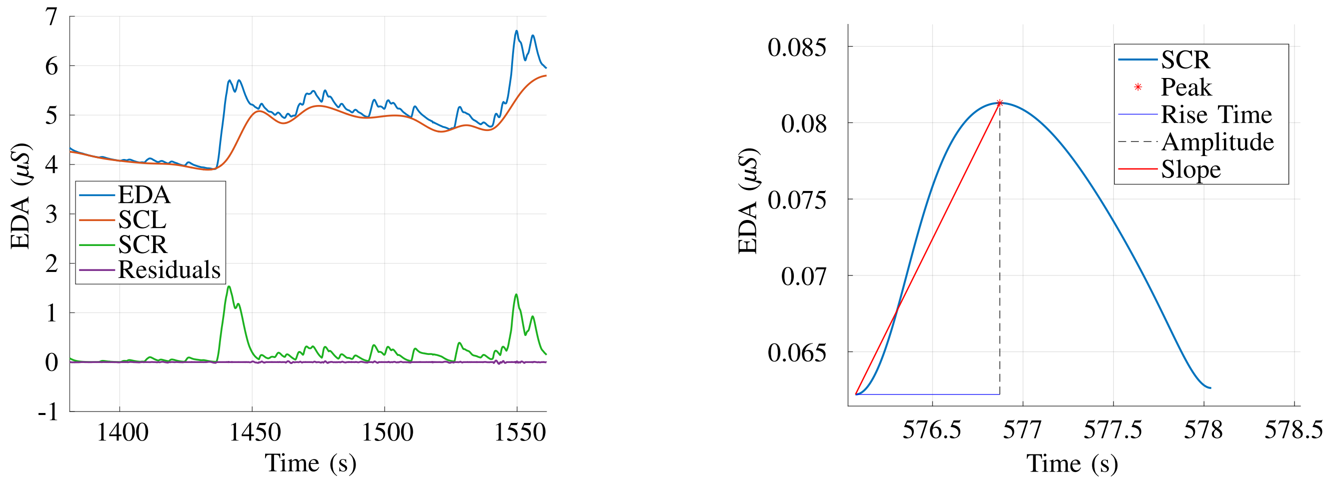

(a) EDA decomposition output.

(b) EDA single wave features.

Fig. 4. Figure 4a provides an example of the output obtained from the EDA signal processing algorithm presented in [12]. The raw EDA in blue is recognizable, the SCL's slow component in green, the SCR's fast component in light blue, and the residuals in red. The SCR's characteristics are described in Figure 4b.

The selected PPG, EDA, and skin temperature features are explained in the following sections. In particular, the processing of the three physiological signals is described separately, and an introduction to the selected characteristics, followed by an explanation of the steps taken in their evaluation, is reported. The considered features related to monitoring the heart, skin, and temperature activities are written in Table II in the Main Manuscript. Finally, it is important to highlight that each signal was divided into each of the fifteen phases presented in the previous section, leading to fifteen different values of each feature for each participant.

1) PPG processing: heart activity was assessed by analysing the PPG signal. Thanks to the well-defined characteristics of this physiological signal, we extracted 19 features during this post-processing part of the experiment. They referred to the *PPG shape*'s characteristics, as reported in Figure 2b, and BPM trend in both frequency and time domain. An example of the BPM trend of a participant during the execution of the test is reported in Figure 3.

The first step in processing the PPG was selecting a single starting point to which subsequent signals of EDA and temperature were aligned to remove noise at the beginning of the recording. To do so, the first 10 s of the PPG registration was filtered with a fourth-order Chebyshev Type I passband filter from 0.5 Hz to 1.5 Hz. The second minimum was identified and chosen as the reference starting point (see Figure 2a).

Then, the PPG raw signal was taken again in order to remove the noise with a fourth-order Chebyshev Type I passband filter 0.5 Hz to 10 Hz; the output was processed through a windowing method with a 10 s length, in which every single wave of the PPG was identified, as shown in Figure 2b, to extract all the features reported in Table II of the Main Manuscript related to the *PPG shape*. In particular, identifying the wave peak represented a key factor because, as a consequent step, the temporal distance between them was evaluated to assess the Inter-Beat Intervals (IBIs). Starting from them, it was possible to delete the outliers and calculate the BPM trend by adopting the following equation:

$$BPM\ (-) = \frac{f_s\ (s^{-1})}{IBI\ (samples)} \times 60\ (s) \qquad (1)$$

where f_s represented the sampling frequency of the test. From the BPM trend, shown in Figure 3, it was possible to obtain the features related to the *HR* and *HRV* in the time domain. The final step of the PPG processing was implementing a frequency analysis of the BPM trend through a Power Spectral Density. In particular, Burg's method was used with windows of 168 heartbeats, from which the features related to the frequency domain analysis of the *HRV* were evaluated.

2) EDA processing: As mentioned in the previous section, the EDA signal can be divided into slow and fast components. The former, named Skin Conductance Level (*SCL* in the following Table), was studied by assessing its mean, standard deviation, and slope for each test phase. The latter, specifically Skin Conductance Response (*SCR*), was assessed by evaluating the average mean and standard deviation of the amplitude and rise time of the SCR's peaks and their average number per phase.

The EDA analysis began with the alignment with the starting point obtained during the PPG analysis described in the previous section. Then, both the noise removal and the separation between SCL and SCR were performed through the cvxEDA method introduced by A. Greco et al. [12], giving us the output shown in Figure 4a. It is possible to observe the original EDA signal in blue, the slow tonic component in red, the fast component in yellow, and the residuals of the process in purple. On the output provided by this algorithm, the *SCL* and *SCR* features were evaluated. In particular, to obtain the phasic component's characteristics, a 90 s window and a seventh-order Butterworth low-pass filter at 5 Hz were implemented.

3) Skin temperature processing: since this physiological signal is not so widely studied in the literature, we decided

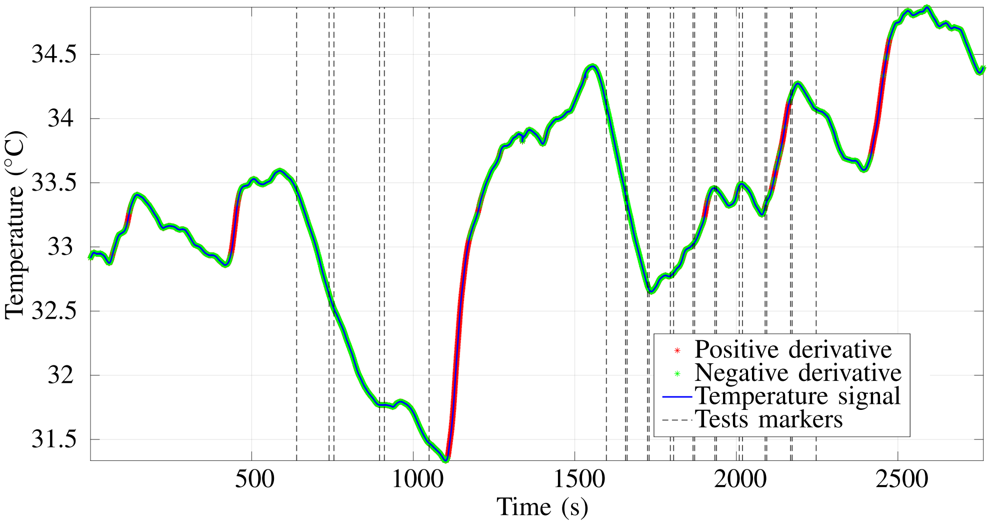

Fig. 5. Example of the raw skin temperature trend, with positive and negative values of the signal's first-time derivative displayed.

to assess some features related to its first derivative, never introduced in the analysis of the MWL and stress level. In particular, the following values were evaluated related to the skin temperature raw signal and its first derivative: initial value, final value, delta (final less initial value), mean, standard deviation, variation over time (delta value over the selected time interval), and variation over time slope (first coefficient of the linear regression of the temperature or its first derivative in the relative phase). As presented in the previous chapter, the first step in processing the skin temperature was represented by the alignment with the aforementioned starting point. To extract all the features, a second-order Butterworth low-pass filter at 10 Hz was realized to remove the noise. In particular, Figure 5 shows a graphical example of the raw signals of the skin temperature and the study about the sign of its first-time derivative.

References

[1] C. Setz et al., "Discriminating stress from cognitive load using a wearable EDA device", *IEEE transactions on information technology in biomedicine : a publication of the IEEE Engineering in Medicine and Biology Society*, vol. 14, no. 2, pp. 410–417, 2010.

[2] H. Posada-Quintero et al., "Innovations in Electrodermal Activity Data Collection and Signal Processing: A Systematic Review.", *Sensors*, vol. 20, no. 2, 2020.

[3] W. -K. Beh et al., "Robust PPG-Based Mental Workload Assessment System Using Wearable Devices," *IEEE Journal of Biomedical and Health Informatics*, vol. 27, no. 5, pp. 2323-2333, May 2023.

[4] P. Ayres et al., "The validity of physiological measures to identify differences in intrinsic cognitive load", *Frontiers in Psychology*, vol. 12, Sep. 2021.

[5] C. Kistler et al., "Fingertip temperature as an indicator for sympathetic responses", *International journal of psychophysiology : official journal of the International Organization of Psychophysiol- ogy*, vol. 29, no. 1, Jun 1998.

[6] Suresh, Abhijit et al. "Prediction of fight or flight response using artificial neural networks." *American Journal of Applied Sciences*, vol 11., pp. 912-920, 2014, 10.3844/ajassp.2014.912.920.

[7] Flavahan, N.A. "Thermoregulation: The Normal Structure and Function of the Cutaneous Vascular System." *Raynaud's Phenomenon*, Springer, New York, NY, 2015, https://doi.org/10.1007/978-1-4939-1526-2_4

[8] R. McCarty, "The Fight-or-Flight Response: A Cornerstone of Stress Research," *Stress: Concepts, Cognition, Emotion, and Behavior*, Academic Press, pp. 33-37, 2016.

[9] F. Scarpina et al., "The stroop color and word test.", *Frontiers in Psychology*, vol. 8, no. 557, Apr. 2017.

[10] N. Janczewski et al., "A meta-analysis of the n-back task while driving and its effects on cognitive workload.", *Transportation Research Part F: Traffic Psychology and Behaviour*, vol. 76, pp. 269–285, Jan. 2021.

[11] N. von Janczewski et al., "A meta-analysis of the n-back task while driving and its effects on cognitive workload", *Transportation Research Part F: Traffic Psychology and Behaviour*, vol. 76, pp. 269-285, 2021, doi.org/10.1016/j.trf.2020.11.014.

[12] A. Greco et al., "cvxeda: A convex optimization approach to electrodermal activity processing", *IEEE Transactions on Biomedical Engineering*, vol. 63, no. 4, pp. 797–804, 2016.
